# A Case of Cutaneous T-cell Lymphoma: The Importance of a Dermatology Consultation

**DOI:** 10.7759/cureus.91416

**Published:** 2025-09-01

**Authors:** Javier A Tobar, Vixey Silva, Vanessa Tan, Krystina Khalil

**Affiliations:** 1 Dermatology, Michigan State University College of Osteopathic Medicine, East Lansing, USA; 2 Dermatology, HCA Florida Largo Hospital, Largo, USA; 3 Dermatology, Larkin Community Hospital Palm Springs Campus, Hialeah, USA; 4 Transitional Year Residency Program, Saint Mary Mercy Hospital, Livonia, USA

**Keywords:** alt-70, cellulitis, cellulitis mimickers, ctcl, cutaneous t-cell lymphoma, telemedicine

## Abstract

Mycosis fungoides (MF), the predominant variant of cutaneous T-cell lymphoma (CTCL), typically manifests as pruritic patches, plaques, and tumors, often mimicking cellulitis. Misdiagnosis of cellulitis has led to substantial avoidable healthcare costs, unnecessary hospitalizations, increased risk of nosocomial infections, and antibiotic resistance. We report a case of cellulitis misdiagnosis in a patient with a history of MF. A 68-year-old male referred from plastic surgery due to suspected worsening of left lower leg cellulitis despite recent antibiotic treatment presented to the hospital. While admitted, the patient underwent a computed tomography scan, which revealed increased diffuse subcutaneous soft tissue changes in the ankle and medial thigh regions with several focal areas suspicious for tumor involvement. The patient received intravenous antibiotics with a plan for surgical debridement and possible amputation per plastic surgery recommendation. Oncology and infectious disease were also consulted as part of the multidisciplinary approach, and a dermatology consultation was recommended, which included a punch biopsy. Results were consistent with an atypical dermal lymphoid infiltrate consistent with CTCL. The patient was discharged with a one-week course of cephalexin and restarted brentuximab per oncology. During follow-up, the patient showed improvement with less erythema and swelling. Delays in CTCL treatment can worsen prognosis. The ALT-70 tool aids in excluding cellulitis. This case underscores the necessity of dermatology consultation for accurate diagnosis, aiming to avert future misdiagnoses and inappropriate treatment of skin conditions resembling cellulitis, such as MF.

## Introduction

In emergency departments (EDs) across the United States from 2010 to 2012, misdiagnosis of lower extremity cellulitis occurred in 30% of cases, leading to 50,000 to 130,000 unnecessary hospitalizations and up to $515 million in avoidable healthcare spending [1]. Antibiotics and hospitalization for misdiagnosed cellulitis are projected to cause more than 9000 nosocomial infections [1]. A review of more than 600 combined articles and case reports related to this dilemma revealed 47 diseases in the differential for lower extremity cellulitis, including lymphoma [2].

Mycosis fungoides (MF), the most common cutaneous T-cell lymphoma (CTCL), is well documented as a “dermatologic masquerader” of many other skin diseases, including cellulitis [3]. MF often develops in sun-protected areas. Lesions initially appear as patches or plaques but may progress to tumor stage in severe cases. However, MF can present in atypical and varied forms, including folliculotropic, ichthyosiform, psoriasiform, and granulomatous [3]. Histologic evidence of epidermotropism, or the presence of T-cells in the epidermis, via skin biopsy helps to ensure the proper diagnosis of MF. While cellulitis is treatable with antibiotics and supportive therapy (e.g., leg elevation) [1,2], MF is not. It is imperative to make the correct, definitive diagnosis to prevent unnecessary antibiotic therapy and avoid delaying treatment, as prolonged time to treatment often worsens patient outcomes.

Dermatologists play a critical role in the healthcare model. Unfortunately, some studies have shown that dermatologists are under-consulted for cellulitis, increasing the rate of misdiagnosis, complications, antibiotic resistance, and financial losses [1]. We present a case of MF that was mistaken for cellulitis in a patient with a known history of MF.

## Case presentation

A 68-year-old male with a chronic and progressive history of MF spanning five years, including folliculotropic and tumor stages, presented to the ED with erythematous and weeping indurated plaques and overlying ulcerations of the left lower leg (LLE). Plastic surgery was consulted, which led to a computed tomography (CT) scan revealing extensive soft tissue ulceration and subcutaneous edema/infiltration, compatible with cellulitis or secondary to infiltrative lymphoma. He was diagnosed with suspected cellulitis and received intravenous vancomycin and cefepime for one day. He was then discharged with a 10-day course of oral doxycycline. However, worsening leg pain and erythema prompted a return to the ED, where further evaluation indicated the potential need for antibiotics, debridement, and amputation.

**Figure 1:**
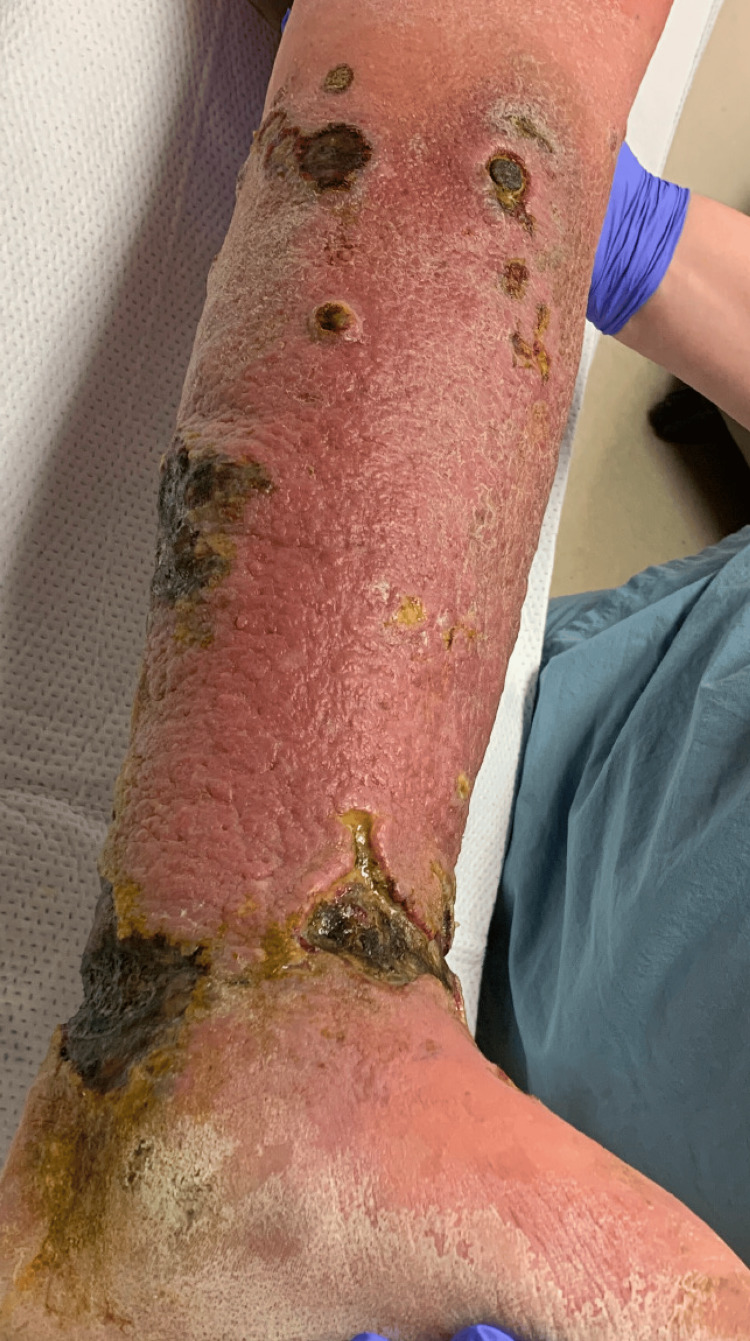
Cutaneous T-cell Lymphoma Taken During Hospital Admission. The figure shows firm edema of the left lower leg and foot with overlying dull red-violaceous erythema with a cobble-stone surface, scant scale, and multiple ulcers of varying sizes with overlying eschar and hemorrhagic crust with undermined borders.

Upon return to the ED, physical examination revealed firm edema of the LLE and foot with dull red-violaceous erythema, a cobblestone surface, and scant scale. Multiple ulcers of varying sizes with overlying eschar and hemorrhagic crust with undermined borders were present, without associated warmth or tenderness to palpation (Figure 1). Repeat CT scan showed diffuse soft tissue changes suggestive of tumor involvement in both the ankle and medial thigh regions, alongside incidental deep vein thrombosis (DVT). Admission ensued with intravenous vancomycin, cefepime, and heparin, pending intervention per plastic surgery for amputation.

The patient ultimately refused to proceed with the planned intervention of amputation. Hematology/oncology suspected infection and chose not to start a chemotherapeutic agent. The cellulitis diagnosis was questioned by Infectious Diseases, which advised a dermatology consultation.

**Figure 2:**
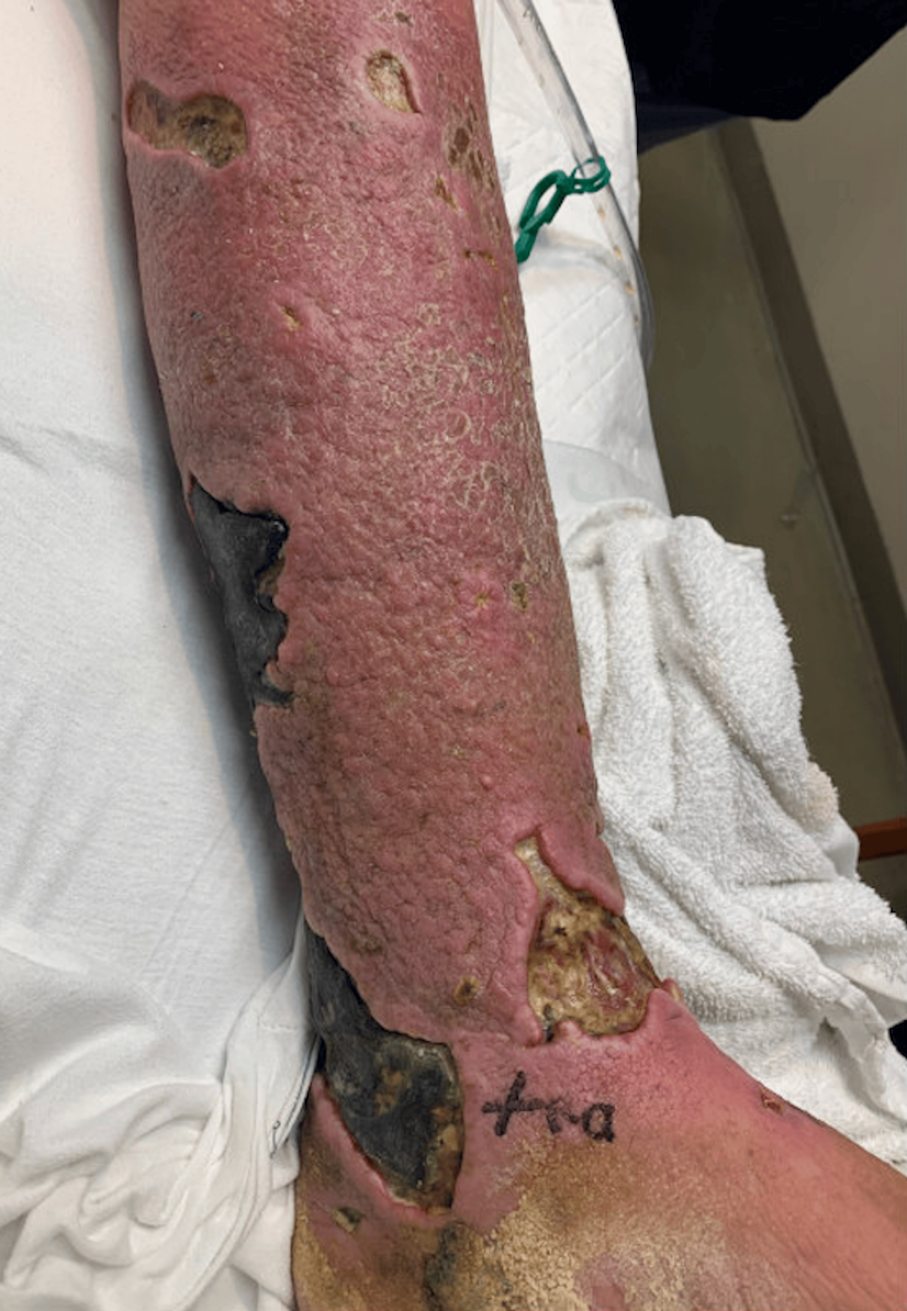
Cutaneous T-cell Lymphoma Taken Approximately One Month After Admission. The figure demonstrates a reduction in the edema and intensity of the erythema, and resolution of the erythema distal to the ankle.

The patient was seen by Dermatology one week into admission, and a punch biopsy was performed to differentiate between CTCL progression and a superimposed infection of CTCL. Biopsy revealed an atypical dermal lymphoid infiltrate consistent with CTCL. On cytologic analysis, atypical cells were determined to be CD3+, CD30+, CD7-, CD4-, and CD8-. Infection was not ruled out by the biopsy, as occasional interstitial neutrophils were present. The patient was discharged with a one-week course of cephalexin and was started on brentuximab per oncology. At both one-week and one-month follow-up, the patient showed improvement with less erythema and swelling (Figure 2). However, the full treatment course of the CTCL was precluded by the patient’s demise secondary to complications thought to be unrelated to this diagnosis.

## Discussion

Tumor-stage MF is biologically aggressive with a poor prognosis. It presents as flat or dome-shaped yellow-red, red-blue, or brown nodules that frequently ulcerate, either alone or in conjunction with eczematous lesions [2,3]. This case highlights a diagnostic challenge, as MF presented with features suggestive of cellulitis, and proper diagnosis prevented unnecessary amputation.

Cellulitis classically presents as a diffuse, bright erythematous, edematous, and warm lesion. It can be poorly demarcated and limited to an area, most commonly a unilateral lower leg [1]. The ALT-70 tool can be used to predict the likelihood of lower extremity cellulitis (Table 1). Scores of five or greater indicate true cellulitis, with positive predictive values of 82-85%. Scores of two or less indicate pseudocellulitis, and alternate diagnoses should be considered. For scores of three or four, a dermatology consultation is recommended [4]. Our patient presented with unilateral lower extremity involvement, a heart rate greater than 90 bpm, and a white blood cell count less than 10,000/µL, yielding a score of four points and indicating a dermatology consultation for appropriate diagnosis. Common cellulitis mimickers include atopic dermatitis, lymphedema, lipodermatosclerosis, irritant dermatitis, and CTCL [1-3]. The lack of warmth, the dull quality of erythema, and the lack of response to antibiotics were essential clues to the true diagnosis in this patient.

**Table 1:** Criteria for ALT-70 Score ED, emergency department; HR, heart rate; WBC, white blood cell. Adapted with permission from ref. [4].

Criterion	Definition	Score
Asymmetry	Unilateral lower extremity involvement	+3
Leukocytosis	WBC count in ED ≥ 10,000/µL	+1
Tachycardia	HR in ED ≥ 90 bpm	+1
Age	≥ 70 years	+2

Dermatologists can play a critical role in the diagnosis and management of cellulitis [5]. However, access to dermatologists in the ED and in inpatient settings can be a challenge for many hospitals. Telemedicine is a potential option that could help address barriers to dermatology access. Store-and-forward teledermatology utilizes patient photographs, which allow dermatologists to perform diagnosis, evaluation, and management of skin disease in the inpatient setting. This method of care is an effective and efficient way of treating patients while also drastically reducing care time and eliminating the distance traveled to deliver care [5].

Our report highlights the importance of dermatology consultations in cases of suspected cellulitis with atypical features. It also demonstrates the value of correct diagnosis, especially in patients with a history of dermatologic disease presenting with new skin findings. There is a critical need for inpatient dermatology services and dermatologists providing these services. We encourage hospitals and dermatologists to consider teledermatology opportunities to overcome the barriers to consultative dermatology services. Dermatologists are best equipped to provide an accurate assessment, diagnosis, and plan when a mimicker of cellulitis (e.g., lymphoma) presents, as seen with our patient.

## Conclusions

This case highlights the diagnostic challenges posed by CTCL, particularly MF, when it mimics cellulitis. CTCL is very difficult to diagnose, even for the most experienced dermatologists, which is why primary care physicians must look for signs and symptoms such as weight loss, swollen lymph nodes, and lesions in sun-protected areas that indicate the need for prompt dermatology consultation. Accurate diagnosis through dermatology consultation and biopsy is crucial to avoid unnecessary treatments and adverse patient outcomes. Misdiagnosis of cellulitis can lead to significant healthcare costs, inappropriate use of antibiotics, and increased risk of nosocomial infections. Dermatologists play a pivotal role in the correct diagnosis and management of skin conditions, and teledermatology offers a promising solution to overcome barriers to specialist access. Familiarizing oneself with the ALT-70 score system is essential, as it aids in differentiating true cellulitis from its mimickers, ensuring timely and accurate diagnosis. Ensuring timely and accurate diagnosis is essential for improving patient care and outcomes in cases of skin conditions that may resemble cellulitis.
